# Clopidogrel discontinuation within the first year after coronary drug-eluting stent implantation: an observational study

**DOI:** 10.1186/1471-2261-14-100

**Published:** 2014-08-13

**Authors:** Troels Thim, Martin Berg Johansen, Gro Egholm Chisholm, Morten Schmidt, Anne Kaltoft, Henrik Toft Sørensen, Leif Thuesen, Steen Dalby Kristensen, Hans Erik Bøtker, Lars Romer Krusell, Jens Flensted Lassen, Per Thayssen, Lisette Okkels Jensen, Hans-Henrik Tilsted, Michael Maeng

**Affiliations:** 1 Department of Cardiology, Aarhus University Hospital, Aarhus, Denmark; 2 Department of Clinical Epidemiology, Aarhus University Hospital, Brendstrupgaardsvej 100, Aarhus, N 8200, Denmark; 3 Department of Cardiology, Odense University Hospital, Odense, Denmark; 4 Department of Cardiology, Aalborg University Hospital, Aalborg, Denmark

**Keywords:** Percutaneous coronary intervention, Dual antiplatelet therapy, Drug-eluting stent, Clopidogrel

## Abstract

**Background:**

The impact of adherence to the recommended duration of dual antiplatelet therapy after first generation drug-eluting stent implantation is difficult to assess in real-world settings and limited data are available.

**Methods:**

We followed 4,154 patients treated with coronary drug-eluting stents in Western Denmark for 1 year and obtained data on redeemed clopidogrel prescriptions and major adverse cardiovascular events (MACE, *i.e.*, cardiac death, myocardial infarction, or stent thrombosis) from medical databases.

**Results:**

Discontinuation of clopidogrel within the first 3 months after stent implantation was associated with a significantly increased rate of MACE at 1-year follow-up (hazard ratio (HR) 2.06; 95% confidence interval (CI): 1.08-3.93). Discontinuation 3-6 months (HR 1.29; 95% CI: 0.70-2.41) and 6-12 months (HR 1.29; 95% CI: 0.54-3.07) after stent implantation were associated with smaller, not statistically significant, increases in MACE rates. Among patients who discontinued clopidogrel, MACE rates were highest within the first 2 months after discontinuation.

**Conclusions:**

Discontinuation of clopidogrel was associated with an increased rate of MACE among patients treated with drug-eluting stents. The increase was statistically significant within the first 3 months after drug-eluting stent implantation but not after 3 to 12 months.

## Background

Dual antiplatelet therapy (DAPT), *i.e.*, aspirin in combination with a P2Y12 antagonist, has been shown to reduce occurrence of ischemic events after coronary stent implantation in randomized clinical trials [1,2]. In real-world settings, however, the level of adherence to recommended DAPT is difficult to assess and its influence on outcomes is less well known. Moreover, the optimal duration of DAPT after coronary stent implantation is disputed [3].

Twelve months of DAPT after percutaneous coronary intervention (PCI) with coronary stent implantation has been recommended in Denmark since November 2002. This recommendation is based on interpretation of results of existing randomized clinical trials [1,2]. In such trials, we expect adherence to treatment to be high owing to patient selection, trial-related follow-up, and cost-free provision of the platelet inhibitor. Adherence to DAPT in real-world settings is most likely lower than in these randomized trials. Assessment of adherence levels and risks associated with discontinuation of DAPT within the first year after coronary stent implantation is needed in real-world settings.

Danish medical registries allow validated monitoring of coronary interventions, prescription redemption, and clinical outcomes [4-9]. Based on data from these registries, we report rates of discontinuation of clopidogrel treatment after coronary drug-eluting stent (DES) implantation and assess the risk of associated adverse events.

## Methods

According to the Central Denmark Region Committees on Health Research Ethics, this study could be conducted without an approval from the Committees.

### Setting

We conducted this population-based cohort study, retrospectively, using medical registries in Western Denmark, which has approximately three million inhabitants (55% of the Danish population). The Danish National Health Service provides universal tax-supported health care, guaranteeing unfettered access to general practitioners and hospitals. Costs of prescription medications, including clopidogrel, are partially reimbursed. Accurate and unambiguous linkage of data from all registries at the individual level is possible in Denmark using the unique central personal registry number assigned to every Danish citizen at birth or upon immigration [9].

### Patients and procedures

We used the Western Denmark Heart Registry (WDHR) [10] to identify all PCI procedures performed between 1 January 2003 and 30 June 2005 [11-13]. For each patient, the first PCI procedure with implantation of one or more coronary DES during the inclusion period was defined as the “index PCI procedure” and the date of that procedure as the “index date”. Patients treated with balloon angioplasty without stenting or with bare metal stents alone were excluded. Only first-generation DES, *i.e.*, sirolimus-eluting (SES) (Cypher, Cordis Corp., Johnson & Johnson, Warren, New Jersey) and paclitaxel-eluting stents (PES) (Taxus, Boston Scientific, Nattick, Massachusetts) were in use during the inclusion period.

The cardiac intervention centers in Western Denmark each perform more than 1,200 PCI procedures per year. The interventions were performed according to current standards including selection of interventional strategy (*e.g.*, pre- or post-dilatation, choice of stent, direct stenting, and administration of periprocedural glycoprotein IIb/IIIa inhibitors).

### Patient characteristics

We obtained information from Danish National Registry of Patients on potential confounders (diabetes and hypertension) between 1977 and the index date [5]. To ensure complete identification of patients with diabetes, we also searched the Danish National Prescription Database for any use of antidiabetic drugs among study participants since 1994 [14]. From the WDHR, we retrieved procedure data, including date of index PCI, indication for PCI (ST-segment elevation myocardial infarction (MI), non-ST-segment elevation myocardial infarction MI or unstable angina pectoris, or stable angina pectoris), number of treated arteries (1, 2, or 3 or more), number of implanted stents (1, 2, or 3 or more), lesion type (A, B1, B2, or C), and stent type.

### Medication use

We used the Danish National Prescription Database to identify all redeemed prescriptions for clopidogrel [14]. Clopidogrel is available only by prescription in Denmark.

The recommended daily maintenance dose of clopidogrel for secondary prevention of ischemic vascular events is 75 mg (one tablet) daily for 12 months following PCI. Thus, for study purposes, the number of days supplied from a redeemed clopidogrel prescription corresponded to the number of tablets in the package. We computed the number of days exposed by adding 14 days to the number of days supplied. This buffer allowed for a 14-day gap to occur between redeemed prescriptions before a patient was considered to have discontinued clopidogrel. This method is well-established [15-17] and a 14-day gap has been used in previous studies [18].

We also identified redeemed prescriptions for aspirin, other nonselective non-steroidal anti-inflammatory drugs, selective cyclooxygenase-2 inhibitors, proton pump inhibitors, calcium channel blockers, statins, vitamin K antagonists, and systemic glucocorticoids.

### Major adverse events

In line with the recommended duration of clopidogrel treatment, we recorded occurrence of major adverse cardiovascular events (MACE) during 12 months after the index date. We defined MACE as the first occurrence of cardiac death, myocardial infarction MI, or definite stent thrombosis (ST). A committee of cardiac specialists, blinded to the history of medication use, reviewed relevant medical records to determine occurrence of definite stent thrombosis ST and cardiac death.

### Cardiac death

We obtained data on all-cause mortality from the Danish Civil Registration System [9]. The committee of cardiac specialists reviewed original death certificates obtained from the National Registry of Causes of Deaths [6]. Deaths were classified as either cardiac or non-cardiac based on the underlying cause recorded on the death certificates. We defined cardiac death as known cardiac death, unwitnessed death, or death from unknown causes.

### Myocardial infarction (MI)

We used the Danish National Registry of Patients to identify hospital admissions with MI as discharge diagnosis [5].

### Stent thrombosis (ST)

Based on review of medical records and angiograms, the committee of cardiac specialists adjudicated the occurrence of definite ST according to Academic Research Consortium definitions [19].

### Statistical analysis

We characterized the patients using medical, procedural, and demographic variables and followed all patients from their index date until date of death or completion of 12 months of follow up.

To describe the pattern of clopidogrel compliance, we first calculated the proportion of patients still alive who had prescription coverage for clopidogrel on each day of follow up. We also calculated the mean and median proportion of days within the first year after stent implantation with prescription coverage for clopidogrel among patients not experiencing MACE.

We then estimated the effect of discontinuing clopidogrel treatment. We defined discontinuation as a gap between prescription redemptions of more than 14 days. We estimated the effect separately according to the time of discontinuation, *i.e.*, within the first 3 months (day 1 through 91), 3 to 6 months (day 92 through 182), or 6 to 12 months (day 183 through 365) after the index PCI procedure. We estimated hazard ratios using a Cox proportional hazards regression model. Hazard ratios were adjusted for potential confounders (age, gender, year of index PCI, indication for PCI, comorbidity level (using Charlson Comorbidity Index scores)) and time-varying use (calculated from the number of days exposed) of aspirin, other nonselective non-steroidal anti-inflammatory drugs, and proton pump inhibitors. Patients entered the study at the time of their first clopidogrel prescription redemption after the index date (delayed entry). Patients contributed to time at risk as current users of clopidogrel as long as they had prescription coverage for clopidogrel. From the time point of discontinuation, patients contributed time at risk according to their time window of discontinuation [15-17].

## Results

The study included 4,154 patients treated with DES. Of these, 2,570 patients were treated with implantation of SES, 1,525 patients with PES, and 59 patients with SES and PES. Patient and procedure characteristics are shown in Table 1.

**Table 1:** Patient and procedure characteristics

	N = 4,154*	**%**
Female	1,127	27.1
Age group (years)		
<60	1,659	39.9
60-70	1,316	31.7
70+	1,179	28.4
Medication use†		
Clopidogrel	3,931	94.6
Proton pump inhibitors	955	23.0
Aspirin	3,718	89.5
Vitamin K antagonists	301	7.2
Nonselective NSAIDs	509	12.3
COX-2 inhibitors	388	9.3
Oral glucocorticoids	309	7.4
Calcium channel blockers	1,153	27.8
Statins	3,606	86.8
Comorbidities‡		
Diabetes	586	14.1
Hypertension	178	4.3
Year of study entry		
2003	847	20.4
2004	1,836	44.2
2005	1,471	35.4
PCI Indication		
STEMI	844	20.3
UAP/NSTEMI	1,277	30.7
SAP	1,902	45.8
Other	131	3.2
Number of treated arteries§		
1	2,877	69.3
2	1,057	25.4
3	212	5.1
Number of Stents§		
1	3,301	79.5
2	631	15.2
3+	208	5.0
Lesion Type§║		
A	898	21.6
B	2,165	52.1
C	1,091	26.3

*Drug-eluting stents only (n = 3,548) and combination of drug-eluting stents and bare metal stents (n = 606).

†Any prescription redemption during follow up.

‡Registered between 1977 and the index PCI date.

§Data were not available on number of treated arteries for 112 patients, on the number of stents for 62 patients, and on lesion type for 6 patients.

║Lesion classification: A = non-complicated, length <10 mm; B = irregular, length 10–20 mm; C = irregular, side branch, 90 degrees, chronic occlusion, length 20 mm.

*Abbreviations*: *COX* cyclooxygenase, *NSAIDs* nonsteroidal anti-inflammatory drugs, *PCI* percutaneous coronary intervention, *STEMI* ST-segment elevation myocardial infarction, *SAP* stable angina pectoris, *UAP* unstable angina pectoris, *NSTEMI* non-ST-segment elevation myocardial infarction.

### Rates of clopidogrel treatment discontinuation

**Figure 1:**
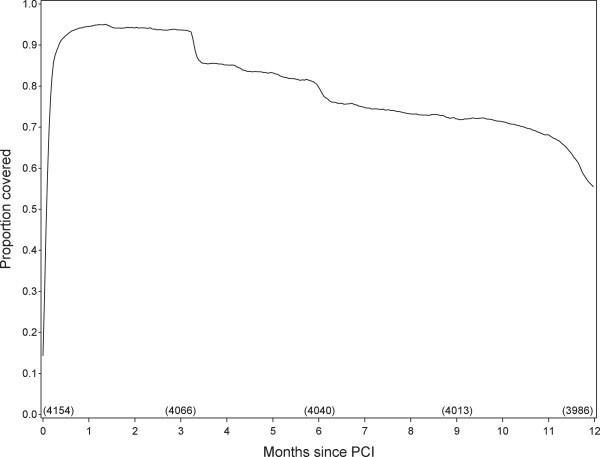
Proportion of patients covered by a prescription for clopidogrel on each day during follow up.

In Figure 1, the proportion of patients with prescription coverage for clopidogrel on each day of follow up is plotted against time since the index PCI procedure date. There was a drop in prescription coverage between 3 and 4 months after the index PCI procedure, and again a smaller drop after about 6 months. The percentage of patients who never redeemed a prescription for clopidogrel during follow up was 5.4%. Among the 3,815 event-free survivors, the mean percentage of days covered by a clopidogrel prescription was 81% (median: 96%).

### Risk associated with clopidogrel treatment discontinuation

**Figure 2:**
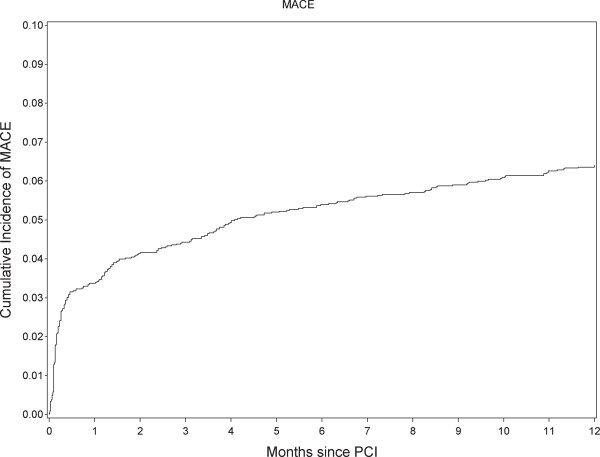
**Cumulative incidence of major adverse cardiac events (MACE).** MACE is a composite of cardiac death, myocardial infarction, and definite stent thrombosis.

**Figure 3:**
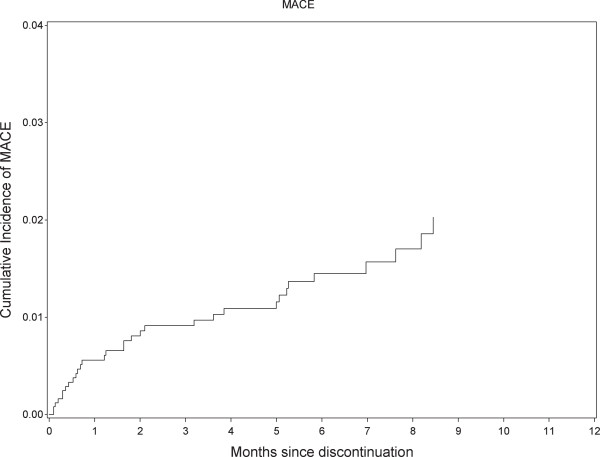
**Cumulative incidence of major adverse cardiac events (MACE) following discontinuation of clopidogrel.** MACE is a composite of cardiac death, myocardial infarction, and definite stent thrombosis.

Figure 2 shows the cumulative incidence of MACE during the first year after the index PCI procedure. The risk of MACE increased most within the first 2 weeks following the procedure (approximately 3%) and then increased more gradually during the remainder of the one-year study period (overall 1-year risk was approximately 6%). Figure 3 shows the cumulative incidence of MACE over time starting from the time-point of discontinuation. The increase in cumulative risk of MACE was highest within the first 2 months following discontinuation.

Table 2 shows crude and adjusted hazard ratios for discontinuation of clopidogrel treatment. The 1-year cumulative MACE rate among patients covered by clopidogrel prescriptions for the full year was 3.9%. Discontinuation of clopidogrel within the first 3 months after PCI was associated with an increased rate of MACE (approximately 2-fold) and cardiac death (almost 5-fold). The risk estimates for the individual components of the combined outcome were also increased, with wider confidence intervals due to fewer events. Definite ST as an individual outcome was too rare within the first 3 months to allow for statistical inference. When discontinuation of clopidogrel occurred later than 3 months following PCI, differences in rates were not statistically significant, however, the hazard ratios suggested that risk of MACE was increased by approximately 30%, cardiac death by 80%, and MI by 10% when clopidogrel was discontinued later than 3 months following PCI. ST was rare and clopidogrel discontinuation between 3-6 months was associated with a non-significant 7-fold higher risk of ST, corresponding to an almost 3% risk of ST in this subgroup.Patients who never redeemed a single clopidogrel prescription were not included in the analyses described above. Among these patients, the cumulative incidence of MACE was 48% within one week, 59% within one month, 61% within 3 months, and 63% within one year following PCI. Thus, these patients experienced very high early MACE rates, but rates after 3 months were comparable to that of the overall patient population, as shown in Figure 2. Early events, such as in-hospital death, may have prevented some of these patients from ever redeeming a clopidogrel prescription.

**Table 2:** Hazard ratios (HRs) for discontinuation of clopidogrel treatment

**Time following DES implantation**		**HR (95% CI)**	**Adjusted HR (95% CI)**
MACE	0-3 months	2.48 (1.30-4.71)	2.06 (1.08-3.93)
	3-6 months	1.26 (0.68-2.33)	1.29 (0.70-2.41)
	6-12 months	1.26 (0.53-2.99)	1.29 (0.54-3.07)
Cardiac death	0-3 months	6.84 (3.06-15.3)	4.80 (2.13-10.8)
	3-6 months	1.95 (0.72-5.30)	1.85 (0.68-5.04)
	6-12 months	1.65 (0.42-6.46)	1.74 (0.45-6.78)
Myocardial infarction	0-3 months	2.05 (0.93-4.53)	1.84 (0.83-4.09)
	3-6 months	1.08 (0.51-2.28)	1.14 (0.54-2.43)
	6-12 months	1.09 (0.36-3.35)	1.09 (0.35-3.34)
Definite stent thrombosis	0-3 months	-	-
	3-6 months	6.04 (0.52-70.2)	7.14 (0.61-83.7)
	6-12 months	-	-

MACE is a composite of cardiac death, myocardial infarction, and definite stent thrombosis.

*Abbreviations*: *HR* hazard ratio, *CI* confidence interval.

Hazard ratios were adjusted for age, gender, year of index PCI, PCI indication, comorbidity level (using Charlson Comorbidity Index scores), and time-varying use (calculated from the number of days exposed) of aspirin, other NSAIDs, and proton pump inhibitors.

0-3 months = day 1 through 91; 3 to 6 months = day 92 through 182; 6 to 12 months = day 183 through 365.

## Discussion

Our main findings from this study of 4,154 consecutive real-world patients treated with first-generation DES are that discontinuation of clopidogrel was common and that discontinuation within the first 3 months after stent implantation was associated with an approximately two-fold increase in risk of MACE and an almost 5-fold increase in risk of cardiac death.

### Registry data

In cohort studies, premature clopidogrel discontinuation after DES implantation is fairly common. Data are conflicting on whether discontinuation, at least beyond the first 4-6 months, is associated with adverse events. These conflicting results may reflect major differences among these studies, including data acquisition, study design, and statistical approach.

Clopidogrel treatment is generally recommended for 12 months after DES implantation. Rates of clopidogrel discontinuation within these 12 months have been reported to be 14% within the first month [20], 28% by 6 months [18], and 4%-38% by 12 months [21-26]. The discontinuation rate reported in our study is similar in magnitude to that of most other reports [18,20-22,25,26].

The timing of clopidogrel discontinuation within the first year appears to be of major importance. Patients discontinuing DAPT within 7, 8-30, or >30 days after PCI due to non-compliance or bleeding had a 7-fold, 2-fold, and 1.3-fold higher risk of MACE, respectively [26]. Among patients with MI, clopidogrel discontinuation within the first month after coronary stent implantation has been associated with a 9-fold increased risk of death by 12 months [20]. Discontinuation within the first year, particularly within the first month, has been associated with adverse events [21]. Based on a landmark approach, 3 studies evaluating event-free patients after 4-6-months of follow-up found that continued use of P2Y12 antagonists did not reduce the risk of adverse events [22,27,28], while 2 other studies showed that it reduced the risk [21,29]. Regional differences, particularly socio-economic factors affecting compliance, may explain this discrepancy in part. In addition, the landmark approach is limited by the healthy survivor effect. Further, in all the studies described above, information about clopidogrel adherence relied on self-reports by patients or their relatives during visits or telephone interviews. Such contacts likely influenced clopidogrel compliance positively, making the data subject to non-differential misclassification bias.

A unique feature of the current study is the linkage of Danish national registries, thereby avoiding the misclassification bias associated with self-reporting. Only a single study previously linked data from a national prescription registry with clinical outcomes to assess the impact of premature clopidogrel discontinuation after DES implantation [18]. Similar to our findings, 28% of study patients discontinued clopidogrel within 6 months after their PCI procedure, and patients who discontinued clopidogrel within the first 6 months had a 2-fold higher risk of death at 12 months. In contrast to this earlier study, the current study included patients younger than 65 years, examined occurrence of MI and ST, and assessed the risk of discontinuing clopidogrel for 3 time intervals.

Individual variation in platelet response to clopidogrel is a subject of debate, providing some of the rationale behind development of newer and currently more expensive platelet inhibitors, such as ticagrelor and prasugrel [30,31]. While the clinical benefits of the newer platelet inhibitors are small compared to clopidogrel, they become significant in large populations of patients with acute coronary syndromes. At the same time, the impact of compliance to DAPT, at least within the first 3 months after PCI, is major. Therefore, approaches to decrease rates of early DAPT discontinuation should be a major focus of future research and daily clinical practice.

### Randomized trial data

Results from 2 on-going clinical trials are awaited [32,33]. Recently, it was reported that 3 months was non-inferior to 12 months of of DAPT after stent implantation [34]. The balance between reduction of ischemic events and the risk of bleeding may favor DAPT for 3 to 6 months after DES implantation and extending DAPT further might be associated with a risk of bleeding outweighing the benefit [35,36]. However, it has been found that DAPT for 1 year was superior to a 1-month regimen [2]. Recently, 4 randomized studies have been conducted, in which event-free patients were randomized to 3 [37], 6 [38,39] or 12 [40] months versus prolonged DAPT following PCI. All 4 trials reported no benefit of prolonged clopidogrel treatment [35,37-40]. Importantly, these randomized data, agree with our registry data based on consecutive real-world patients.

### Limitations

The strength of this study lies in its use of population-based registries with complete follow-up. However, a number of limitations should be acknowledged. First, some patients may have redeemed their prescriptions without taking all or any of the tablets. Moreover, temporary discontinuations < 14 days were not detected. However, others have shown that such short discontinuations do not affect outcomes [24]. Second, aspirin use could not be completely assessed in this study, as aspirin is available without a prescription. This limitation may be relevant at least for ST [22]. However, low-dose aspirin used for secondary prevention of cardiovascular disease generally is prescribed by physicians because prescription costs are partly reimbursed through the national health insurance program. Third, despite adjustment for comorbidities, we cannot exclude residual confounding. Fourth, it has been reported that the positive effect of compliance with medication regimens is lower than estimated from observational studies [41]. The true protective effect of clopidogrel in routine clinical care patients thus may be lower than estimated here. Fifth, the decline in adherence to clopidogrel treatment after 3-4 months and 6 months after the index PCI procedure was likely caused by a combination of medical decisions for discontinuation and patients’ lack of compliance [20,23,26]. Sixth, the results obtained are limited to the 2 first-generation DES. As newer DES are considered safer than the first generation DES [42], the impact of discontinuation of prolonged DAPT is likely to be lower with newer stents.

## Conclusion

Discontinuation of clopidogrel was associated with an increased rate of MACE among patients treated with DES. The increase was statistically significant within the first 3 months after DES implantation but not after 3 to 12 months.

## Abbreviations

CI: Confidence interval; HR: Hazard ratio; MACE: Major adverse cardiovascular events; PCI: Percutaneous coronary intervention; WDHR: Western Denmark Heart Registry.

## Competing interests

TT: Teaching honorarium from AstraZeneca. Travel grants from St. Jude Medical and The Medicines Company. MBJ, GEC, MS, HTS, LT, HEB, LRK, JFL, PT: None. SDK: Lecture fees from AstraZeneca, BMS, Eli Lilly, and The Medicines Company. AKK: Speakers fee from Cordis and St Jude Medical. Travel grants from Abbott, Biotronik, Cordis, Medtronic, Terumo, The Medicines Company, and St. Jude Medical. LOJ: Unrestricted grant from Terumo and honoraria from AstraZeneca. HHT: None. MM: Travel grants from Abbott, St. Jude Medical, Medtronic, Biotronik, and Terumo.

## Authors’ contributions

TT conception and design, data quisition, analysis and interpretation of data, and drafting of manuscript. MBJ: analysis and interpretation of data, and drafting of manuscript. GEC, MS, LT, HEB, SDK, LRK, AKK, JFL, PT, LOJ, HHT data quisition and review of manuscript. HTS conception and design and review of manuscript. MM conception and design, data quisition, analysis and interpretation of data, drafting and review of manuscript. All authors read and approved the final manuscript.

## Pre-publication history

The pre-publication history for this paper can be accessed here:

http://www.biomedcentral.com/1471-2261/14/100/prepub
